# Tucum-Do-Cerrado (*Bactris setosa* Mart.) Consumption Modulates Iron Homeostasis and Prevents Iron-Induced Oxidative Stress in the Rat Liver

**DOI:** 10.3390/nu8020038

**Published:** 2016-02-17

**Authors:** Adriana M. Fustinoni-Reis, Sandra F. Arruda, Lívia P. S. Dourado, Marcela S. B. da Cunha, Egle M. A. Siqueira

**Affiliations:** 1 Department of Nutrition, Faculty of Health Sciences, Campus Universitário Darcy Ribeiro, Universidade de Brasília, 70.910-900 Brasília, DF, Brazil; adriana.fustinoni3@gmail.com (A.M.F.-R.); liviapsdourado@gmail.com (L.P.S.D.); desa.marcela@gmail.com (M.S.B.C.); 2 Department of Cell Biology, Biological Sciences Institute, Campus Universitário Darcy Ribeiro, Universidade de Brasília, 70.910-900 Brasília, DF, Brazil; eglemasi@gmail.com

**Keywords:** oxidative stress, tucum-do-cerrado (*Bactris setosa* Mart.), Hamp, Nfr2

## Abstract

This study investigated the effect of tucum-do-cerrado consumption in the oxidative status of iron-supplemented rats. Four groups of rats were treated: Control (AIN-93G), Tuc (AIN-93G added of tucum-do-cerrado), Fe (AIN-93G iron-enriched), or TucFe (AIN-93G with tucum-do-cerrado and iron-enriched) diet, for 30 days. Iron-enriched diet increased serum, liver, spleen, and intestine iron levels; transferrin saturation; liver lipid oxidation; mRNA levels of hepatic Hamp and Bmp6, and Nrf2 in the intestine. Tucum-do-cerrado consumption reduced spleen lipid and protein oxidation; mRNA levels of hepatic Hamp and Ftl, and increased serum antioxidant capacity and hepatic mRNA levels of Bmp6, Hmox1, Nqo1, and Nrf2. TucFe diet consumption abrogated the liver Hamp iron-induced up-regulation, prevented intestinal iron accumulation; hepatic lipid peroxidation; splenic protein damage, and the increase of catalase, glutathione reductase, and glutathione peroxidase activity in some tissues. These results suggest that tucum-do-cerrado protects tissues against oxidative damage, by reducing iron availability in liver and consequently inhibiting liver Hamp expression.

## 1. Introduction

Iron is an essential nutrient involved in several biological functions such as oxygen transport, oxidative metabolism of nutrients for energy production, erythropoiesis and as a co-factor of antioxidant enzymes [1]. As a transition metal, iron also acts as a pro-oxidative ion by catalyzing the conversion of weakly-reactive oxygen species (ROS) such as H_2_O_2_ into highly reactive hydroxyl radicals, which in turn promotes oxidative stress in cells [2]. The accumulation of cellular oxidative damage is associated with several chronic diseases and premature aging, which possibly can be related to iron overload [3,4,5]. In response to this adverse effect, mammals employ distinct mechanisms to regulate iron homeostasis: at cellular level, by a molecular mechanism that involves iron regulatory proteins and iron responsive elements (IRP/IRE) and at systemic level, by the hepatic hormone hepcidin. Levels of hepcidin regulate the uptake and exportation of iron by enterocytes and macrophages, respectively, which orchestrate tissue iron levels and mobilization. In turn, the body iron status regulates the synthesis of hepcidin hormone, by the hemojuvelin/Bmp6 pathway which adjusts the body’s iron demands through iron mobilization and intestinal absorption by regulating the level of ferroportin protein [1]. The hepcidin-ferroportin complex is internalized into the cell where ferroportin is degraded, resulting in the reduction of cellular iron exportation into the bloodstream [6,7]. This regulatory mechanism avoid iron body overload and, consequently, reduces iron-induced oxidative stress. In addition to the iron regulatory mechanism, mammals also have established an efficient antioxidant mechanism by counterbalancing endogenous ROS production that comprises antioxidant enzymes and non-enzymatic antioxidant defenses such as glutathione that are involved in scavenging ROS [8,9]. Increased oxidative damage can induce the gene expression of antioxidant and detoxifying proteins, once these genes present an antioxidant response element (ARE), which is a specific nucleotide sequence present in the promoter regions of the genes. The transcription factor nuclear factor-erythroid 2-related factor 2 (Nrf2) binds to ARE and regulates the expression of antioxidant enzymes, such as catalase, heme oxygenase-1, and NAD(P)H dehydrogenase quinone 1, among other genes involved in antioxidant defenses [10,11]. Some recent studies demonstrated that iron supplementation activates hepatic NRF2 and consequent increases the expression of NRF2-regulated cytoprotective genes and NRF2 target proteins, protecting cells from the toxic effects of iron excess [12,13]. An imbalance between antioxidants and oxidants in favor of oxidants, promotes oxidative stress in cells.

In addition to these endogenous antioxidant defenses and strengthening the antioxidant cellular system, foods, especially vegetables, contribute to a variety of antioxidant molecules that can protect animal cells against oxidative stress [14]. A biome known as Cerrado dominates the central region of Brazil and houses enormous biodiversity with numerous (and little-studied) endemic species [15,16,17]. In a previous study performed in our laboratory, we compared the concentration of phenolic compounds and antioxidant activities (AA) in the edible parts of twelve Cerrado plants species. We identified at least seven Cerrado plant species with higher antioxidant potential relative to the Red Delicious apple [18]. Among these species, the tucum-do-cerrado (*Bactris setosa* Mart.), a fruit with a purple peel, whitish pulp, and a unique and large seed that is produced by a palm tree, was one of the highlighted fruits. Considering the high antioxidant activity of tucum-do-cerrado extracts *in vitro* and that iron accumulation in tissues may be associated to the accumulated oxidative damage, this study investigated the effect of tucum-do-cerrado consumption on oxidative stress induced by dietary iron supplementation and the relationship between the antioxidant potential of tucum-do-cerrado and the expression of genes involved in iron homeostasis, in rats.

## 2. Experimental Section

### 2.1. Tucum-Do-Cerrado Fruit

Tucum-do-cerrado fruit (Family: *Arecacea*; Genus: *Bactris* and Species: *Bactris setosa* Mart.) samples were collected in the harvested season (from January to March) at full maturity, at a farm localized at Terezópolis de Goiás, 16°28′15.4″ S and 49°03′44.1″ W, Goiás, Center-West Region of Brazil. A subsample of the specimen was deposited in the UB Herbarium of University of Brasília, Brazil, with an identification number 124364. The permission to collect was issued by the *Instituto Brasileiro do Meio Ambiente e dos Recursos Naturais Renováveis* (IBAMA)/*Ministério do Meio Ambiente* (Authorization Number 9/2012, IBAMA/Ministério do Meio Ambiente). The tucum-do-cerrado fruits were washed with distilled water and stored at −80 °C until diet preparation. After manually removing the seeds from frozen fruits, the pulp and peel were homogenized in a blender with a small amount of water, added and homogenized in the rats’ diets, after homogenization diets were pelletized.

### 2.2. Animals’ Treatment

Twenty-four male 21-day-old Wistar rats were provided by Granja Roberto Giannichi, (Suzano, São Paulo, Brazil). They were housed individually in stainless steel cages at a temperature of 21.75 ± 0.46 °C under a 12-h light/dark cycle. The rats had free access to purified water and food during the dark cycle. The rats were maintained on AIN-93G diet until they reach adulthood, an average weight of 250 g. After 37 days of acclimatization, the rats (278.9 ± 20 g) were randomly assigned into four groups, (6 rats/group) as follows: the control group (CNT) received a rodent diet, the AIN-93G diet, containing 35 mg of iron/kg diet [19]; the iron-enriched group (Fe) was fed the AIN-93G diet containing 350 mg of iron/kg diet; the tucum-do-cerrado group (Tuc) received the AIN-93G diet with 150 g of tucum-do-cerrado fruit (pulp and peel)/kg diet; and the tucum-do-cerrado iron-enriched group (TucFe) received iron-supplemented rodent diet (350 mg of iron/kg diet) and 150 g of tucum-do-cerrado fruit/kg diet (the concentration of tucum-do-cerrado provided to the animals was estimated based on the recommendation of five servings of fruits per day for an healthy adult individual, adjusted to the animal weight). The iron concentration of the iron-supplemented diet was ten times higher than the iron recommendation for rodents (35 mg/kg diet); this value was established based on the iron supplementation dose for adult humans (80 mg Fe/day), which is about ten times higher than the value of the Recommended Dietary Intake (RDA = 8 mg/day for males 19–50 years).

The AIN-93G diet was modified for groups that received tucum-do-cerrado fruit to adjust the macronutrient compounds to rat requirements (Supplementary Material Table S1). The rats were weighed weekly, and their food intake was recorded daily. After 30 days of treatment, the rats were anesthetized in an anesthetic chamber with isoflurane (Cristália, Itapira, São Paulo, Brazil) and sacrificed by cardiac puncture. Blood samples were collected into two tubes with and without 7.0% EDTA (21 µL/mL blood) and the organs, specifically the liver, spleen, kidney, and intestine (a 1 cm length of the small intestine distal to the pylorus and 1 cm proximal to the ileocecal valve) was excised and the lumen was rinsed with saline, were excised, washed in a cold 0.9% NaCl solution, rapidly frozen in liquid nitrogen and stored at −80 °C for further analysis. The present study was approved by the Animal Ethics Committee of the Biological Sciences Institute, University of Brasília (UnBDOC N° 120380/2009), following the Brazilian Guidelines for Care and Use of Animals for Scientific Purposes and Teaching—normative resolution number 12 of 20 September 2013—of the National Council for the Control of Animal Experimentation.

### 2.3. Tissue Iron Determination and Serum Iron Parameters

The iron concentrations in the tissues were determined by using a method described by Baranowska, *et al.* [20]. Serum iron, unsaturated iron binding capacity (UIBC), total iron binding capacity (TIBC), transferrin saturation (TS), and transferrin concentration were analyzed by using a colorimetric kit, as described in the manufacturer’s Labtest Diagnóstica S.A. (Lagoa Santa, Minas Gerais, Brazil).

### 2.4. mRNA Expression of Iron Metabolism and Antioxidant Genes

#### 2.4.1. RNA Extraction and cDNA Synthesis

Total RNA extraction of the liver, spleen, kidney, and intestine samples was performed with Trizol reagent (Invitrogen, Carlsbad, CA, USA), according to the manufacturer protocol. RNA was quantified using spectrophotometry (Shimadzu spectrophotometer—TCC 240A) at 260 nm and tested for purity (by A260/280 ratio and A260/230 ratio) and integrity (by denaturing gel electrophoresis). The complementary DNA (cDNA) synthesis was performed using the Reverse Transcription Kit Improm II System (Promega, Madison, WI, USA).

#### 2.4.2. mRNA Quantification by Real-Time Polymerase Chain Reaction

Real-time PCR was performed by using the Fast SYBR Green Master Mix 2X reagent (Applied Biosystems, Foster City, CA, USA) with 2 µL of cDNA (corresponding to 0.2 ng of total RNA) in a final volume of 10 µL with 5 µL Fast SYBR Green Master Mix and 0.2 µmol/L (final concentration) of each primer. The primer sequences are listed in Supplementary Material (Table S2). Quantitative PCR was performed with a 7500 Fast Real-Time PCR System (Applied Biosystems, Foster City, CA, USA) for 40 cycles at 95 °C for 20 s, 60 °C for 3 s and 60 °C for 30 s. The comparative C_T_ method was used to quantify the abundance of target gene mRNA, and the results are presented as 2^−∆∆CT^ [21]. The relative expression of the genes was normalized to β-actin mRNA levels.

### 2.5. Antioxidant Enzyme and NADPH Oxidase Tissue Activity

The activity of catalase (CAT, EC 1.11.1.6), glutathione reductase (GR, EC 1.6.4.2), glutathione reductase (GR, EC 1.6.4.2), glutathione peroxidase (GPx, EC 1.11.1.9), glutathione peroxidase (GPx, EC 1.11.1.9), glutathione-S-transferase (GST, EC 2.5.1.18), and NADPH Oxidase (Nox, EC 1.6.3.1) was assessed in the liver, spleen, intestine, and kidney as previously described [4].

### 2.6. Tissue Lipid Peroxidation Level

The malondialdehyde (MDA) concentrations in liver, spleen, intestine, and kidney homogenates were measured by high performance liquid chromatography (25 cm Shim-park C18 CLC-ODS (M) column Shimadzu, Kyoto, Japan) [22]. The spectrofluorometric detector wavelengths were set at 532 nm (excitation) and 553 nm (emission). A four-point standard curve (0.05–2.02 nmol/mL) was produced with tetraethoxypropane (TEP, Sigma, St. Louis, MO, USA) dissolved in 1% H_2_SO_4_ because the acid hydrolysis of TEP yields stoichiometric amounts of MDA (*y* = 10^−6^*x* + 0.0203; *r*^2^ = 0.9998). The total protein concentrations of the homogenates were determined by using a method by Hartree [23]. The results were expressed as nmol MDA/mg total protein.

### 2.7. Tissue Protein Oxidation Level

The protein oxidation of liver, spleen, intestine, and kidney homogenates was assessed by carbonyl content according to a method by Richert, *et al.* [24]. The absorbance was measured at 376 nm (spectrophotometer Shimadzu—TCC 240A) and the carbonyl content was expressed as the nmol of carbonyl groups per milligrams of total protein with an extinction coefficient of 22 mmol∙L^−1^∙cm^−1^. The total homogenate protein concentration was determined according to Hartree [23].

### 2.8. Antioxidant Capacity of Serum (FRAP)

The antioxidant capacity of serum was estimated via FRAP assay according to Benzie and Strain [25], with modifications.

### 2.9. Statistical Analysis

Comparisons among treatments were tested using One-Way ANOVA test with Bonferroni correction, using SPSS version 17 software (SPSS Inc., Chicago, IL, USA). The level of statistical significance was set at *p* < 0.05. All values are expressed as the mean ± standard deviation (SD).

## 3. Results

### 3.1. Effect of Tucum-Do-Cerrado Consumption on Food Intake, Iron Intake, Body Weight Gain and Iron Concentration in the Tissues

**Figure 1:**
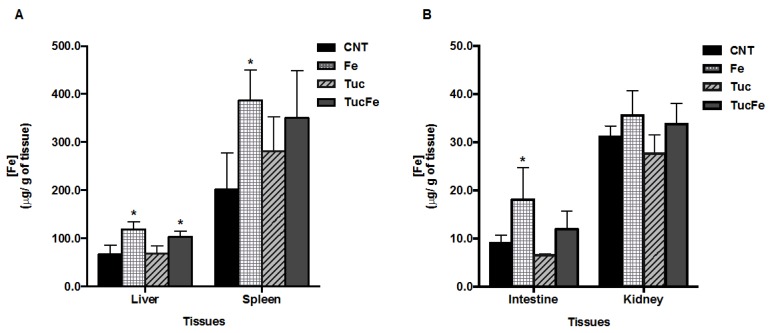
Iron levels in tissues, according to group treatment. CNT, Control group; Fe, iron-enriched group, Tuc, tucum-do-cerrado group; TucFe, tucum-do-cerrado iron-enriched group. Data are the means ± SD (*n* = 6). Significant differences (*p* < 0.05): * Comparison to the Control group.

After 30 days of treatment, there was no difference in the weight gain and dietary intake among the groups (Table 1). As expected, Fe and TucFe rats showed the highest intake of iron compared to control rats (*p* < 0.001 for both). The Fe group showed higher iron concentration in the liver, spleen, and intestine, compared to the control (*p* < 0.001, 0.019 and 0.013, respectively; Figure 1). Moreover, the Fe group showed an increase in serum iron and transferrin saturation (TS) relative to the control group (*p* = 0.002 for both, Table 2). The Tuc group showed iron intake similar to the control group; and tucum-do-cerrado did not change the iron levels in all studied tissues including the serum, compared to the control group (Figure 1). The iron concentrations in the spleen and in the intestine from iron-supplemented rats treated with tucum-do-cerrado (TucFe group) was not different from those of the control rats (Figure 1). The iron-supplemented rats treated with tucum-do-cerrado (TucFe group) showed no significant difference in tissue iron levels compared to the iron supplemented rats (Fe group). However, serum iron levels and transferrin saturation of TucFe group were lower than the Fe group (*p* < 0.001 for both groups, Table 2).

**Table 1:** Effects of tucum-do-cerrado (*Bactris setosa* Mart.) consumption on dietary intake and the body weight gain of rats treated with AIN-93G diet supplemented or not supplemented with iron for 30 days.

**Dietary Intake (g)**	**CNT**	**Fe**	**Tuc**	**TucFe**
	573.9 ± 36.1	512.8 ± 48.1	571.5 ± 56.5	584.1 ± 35.6
**Body Weight Gain (g)**	104.5 ± 19.2	105.2 ± 7.4	82.4 ± 10.8	97.8 ± 24.0
**Iron Intake (mg)**	18.8 ± 1.0	117.0 ± 11.0 *	24.1 ± 2.4	123.9 ± 7.6 *
**Tucum-Do-Cerrado Intake (g)**	−	−	66.8 ± 6.6 *	68.2 ± 3.7 *^,**#**^

Control group (CNT); iron-enriched group (Fe); tucum-do-cerrado group (Tuc); tucum-do-cerrado iron-enriched group (TucFe). Data are means ± SD (*n* = 6). Significant differences (*p* < 0.05): * Comparison to the Control group; ^#^ Comparison to the Fe group.

**Table 2:** Effects of tucum-do-cerrado (*Bactris setosa* Mart.) consumption on serum iron parameters in rats treated with AIN-93G diet supplemented or not supplemented with iron for 30 days.

	CNT	Fe	Tuc	TucFe
**Serum Iron (µg/dL)**	103.4 ± 42.2	222.7 ± 53.3 *	128.5 ± 31.5	74.8 ± 13.3 ^#^
**UIBC (µg/dL)**	266.0 ± 93.0	248.8 ± 71.3	276.0 ± 43.5	310.3 ± 15.8
**TIBC (µg/dL)**	403.9 ± 24.9	438.4 ± 73.6	397.7 ± 50.7	385.1 ± 17.4
**Transferrin Saturation (%)**	25.9 ± 11.7	50.7 ± 8.6 *	32.3 ± 6.9	19.4 ± 3.1 ^#^
**Transferrin (mg/dL)**	282.7 ± 17.4	307.0 ± 51.5	278.4 ± 35.5	269.6 ± 12.2

Control group (CNT); iron-enriched group (Fe); tucum-do-cerrado group (Tuc); tucum-do-cerrado iron-enriched group (TucFe). Data are means ± SD (*n* = 6). Significant differences (*p* < 0.05): * Comparison to the Control group; ^**#**^ Comparison to the Fe group.

### 3.2. Effect of Tucum-Do-Cerrado Consumption on the Oxidative Damage to Lipid and Protein in Tissues

**Figure 2:**
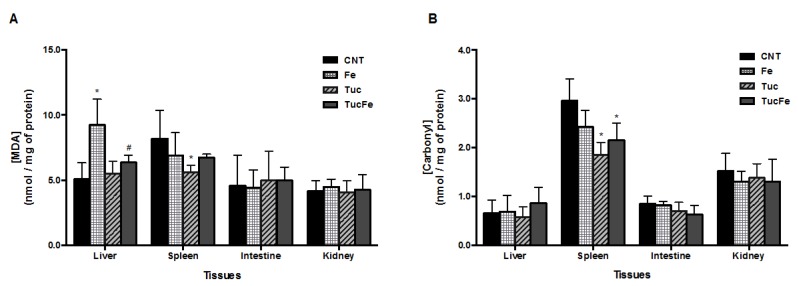
Levels of malondialdehyde (MDA) and carbonyl protein in the tissues, according to group treatment. CNT, Control group; Fe, iron-enriched group, Tuc, tucum-do-cerrado group; TucFe, tucum-do-cerrado iron-enriched group. Data are the means ± SD (*n* = 6). Significant differences (*p* < 0.05): * Comparison to the Control group; ^#^ Comparison to the Fe group.

The dietary iron supplementation for 30 days significantly increased the level of malondialdehyde (MDA) in liver of Fe group relative to the control group (*p* < 0.001). The consumption of tucum-do-cerrado by iron-supplemented rats (TucFe) significantly decreased the level of MDA in liver relative to the Fe group (*p* = 0.013; Figure 2). Lipid peroxidation (MDA) was also reduced in the spleen of the Tuc rats in relation to the control rats *(p* = 0.044). Carbonyl levels were lower in the spleen of the rats that had received diets added of tucum-do-cerrado, Tuc and TucFe groups, relative to the control group (*p* = 0.001 and 0.014; Figure 2).

### 3.3. Effect of Tucum-Do-Cerrado Consumption on the Activity of Antioxidant Enzymes and on Serum Antioxidant Capacity in Tissues

**Figure 3:**
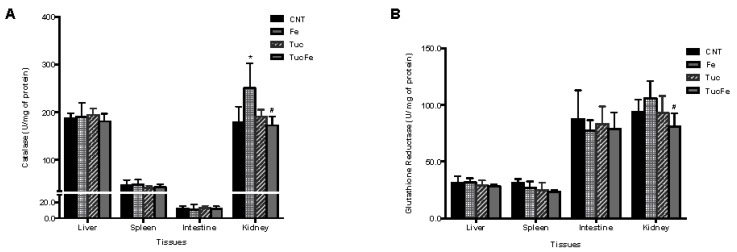

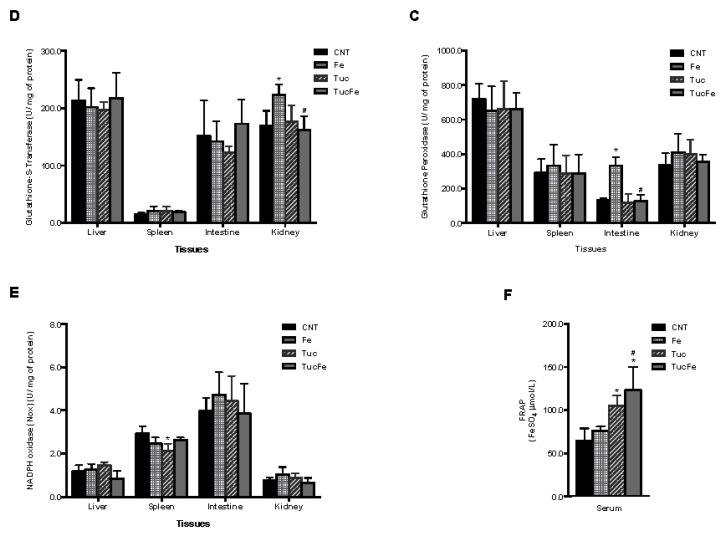
Specific activity of catalase (**A**); glutathione reductase (**B**); glutathione peroxidase (**C**); glutathione-S-transferase (**D**); NADPH oxidase (**E**) in tissues homogenates, and serum antioxidant capacity (**F**), according to group treatment. CNT, Control group; Fe, iron-enriched group, Tuc, tucum-do-cerrado group; TucFe, tucum-do-cerrado iron-enriched group. Data are the means ± SD (*n* = 6). Significant differences (*p* < 0.05): * Comparison to the Control group; ^#^ Comparison to the Fe group.

The oxidative status of rats was measured by the determination of the enzymatic activities of catalase (CAT), glutathione reductase (GR), glutathione peroxidase (GPX), glutathione-s-transferase (GST), and NADPH oxidase (Nox) and the results are presented in Figure 3. The iron-enriched diet significantly increased the specific activity of catalase (CAT) and glutathione-s-transferase (GST) in the kidney and glutathione peroxidase (GPx) in the intestine relative to the control group (*p* = 0.016; 0.017 and <0.001, respectively; Figure 3). Tucum-do-cerrado consumption did not change the specific activities of the analyzed antioxidants enzymes in the Tuc group. However, the consumption of tucum-do-cerrado by the iron-supplemented group (TucFe) significantly reduced the CAT, glutathione reductase (GR) and GST activities in the kidney and GPx in the intestine, in comparison with the Fe group (*p* = 0.009; 0.039; 0.006 and <0.001, respectively). The serum antioxidant capacity (measured by FRAP assay) was significantly higher in the rats treated with tucum-do-cerrado diet (Tuc) and even in those receiving an iron-enriched diet (TucFe) relative to the control (*p* = 0.026 and 0.001, respectively; Figure 3). A positive effect of tucum-do-cerrado consumption was observed on the serum antioxidant capacity in TucFe rats relative to the Fe rats (*p* = 0.008; Figure 3).

### 3.4. The Effect of Tucum-Do-Cerrado Consumption on the mRNA Levels of Proteins Involved in Body Antioxidant Capacity: Nrf2, Cat, Hmox1, and Nqo1

**Figure 4:**
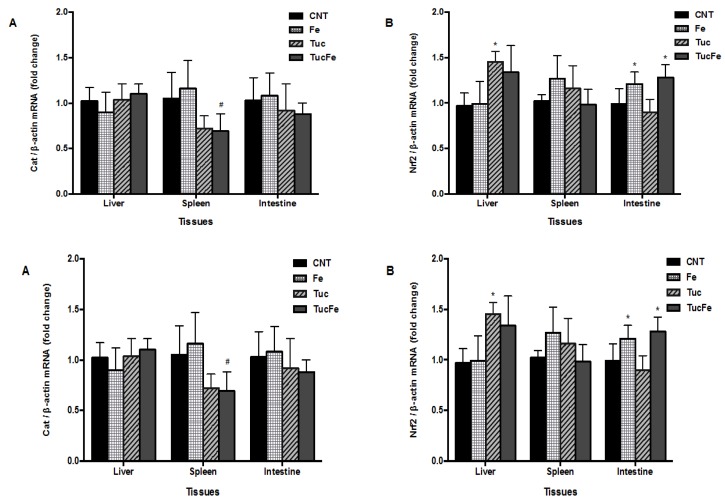
Quantification of mRNA levels of nuclear factor erythroid 2-related factor 2 (Nrf2) and catalase (Cat) in the liver, spleen and intestine and heme oxygenase-1 (Hmox1) and NAD(P)H dehydrogenase quinone 1 (Nqo1) in the liver, determined by qPCR. CNT, Control group; Fe, iron-enriched group, Tuc, tucum-do-cerrado group; TucFe, tucum-do-cerrado iron-enriched group. Data are the means ± SD (*n* = 6). Significant differences (*p* < 0.05): * Comparison to the Control group; ^#^ Comparison to the Fe group.

Figure 4 shows the relative transcript levels of nuclear factor erythroid derived 2 like 2 (Nfe2l2, Nrf2) and catalase (Cat) in rat livers, spleens, and intestines, and of hepatic heme oxygenase-1 (Hmox1) and NAD(P)H dehydrogenase quinone 1 (Nqo1), normalized to values obtained for β-actin (Actb). The group treated with tucum-do-cerrado showed an increase of Nrf2 mRNA levels in the liver relative to the control group (*p* = 0.012; Figure 4). In the spleen, no difference was obtained in the Nrf2 mRNA levels among all treatment groups. Iron supplementation increases Nrf2 mRNA levels in the intestine in the presence or not of tucum-do-cerrado consumption, in relation to control group (*p* = 0.016 and 0.035, respectively; Figure 4). The catalase mRNA levels were lower in the spleen of rats that received iron supplementation and tucum-do-cerrado (TucFe group) compared to the Fe group (*p* = 0.025); however, no change was found in the catalase activity of the spleen among the groups (Figure 3). In relation to Hmox1 and Nqo1 transcripts levels, the group fed the tucum-do-cerrado diet (Tuc) showed an increase in liver Hmox1 and Nqo1 mRNA levels relative to the control group (*p* = 0.008 and *p* < 0.001, respectively).

### 3.5. The Effect of Tucum-Do-Cerrado Consumption on the mRNA Levels of Hamp in the Liver

**Figure 5:**
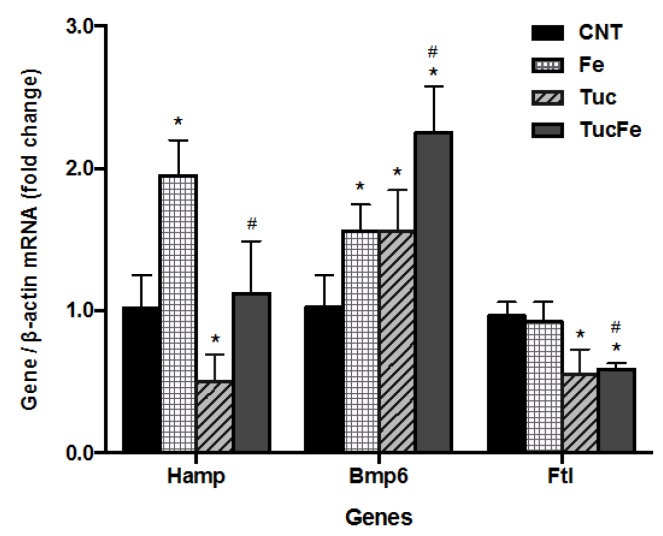
Quantification of hepatic mRNA levels of hepcidin (Hamp), bone morphogenetic protein 6 (Bmp6) and ferritin (Ftl), determined by qPCR. CNT, Control group; Fe, iron-enriched group, Tuc, tucum-do-cerrado group; TucFe, tucum-do-cerrado iron-enriched group. Data are the means ± SD, (*n* = 6). Significant differences (*p* < 0.05): * Comparison to the Control group; ^#^ Comparison to the Fe group.

As expected, at the end of the experimental period, the iron-supplemented group (Fe group) showed an increase in liver Hamp mRNA levels compared to control group (*p* < 0.001, Figure 5). Surprisingly, the addition of tucum-do-cerrado to the diet (Tuc group) reduced liver Hamp mRNA levels relative to the control group (*p* = 0.026), and the reduction was also observed by adding tucum-do-cerrado to the iron-enriched diet (TucFe group) relative to the Fe group (*p* < 0.001), making this value no different from the control group.

### 3.6. The Effect of Tucum-Do-Cerrado Consumption on the mRNA Levels of Bmp6 and Ftl in the Liver

To clarify the effect of tucum-do-cerrado on the modulation of iron homeostasis, it was determined the mRNA levels of Bmp6 and Ftl in the liver. All test groups (Fe, Tuc, and TucFe) showed an increase of liver Bmp6 mRNA levels compared to control group (*p* = 0.017, 0.023 and <0.001, respectively; Figure 5). The TucFe group also showed an increase of Bmp6 mRNA levels compared to Fe group (*p* = 0.003). A reduction in liver Ftl mRNA levels was observed in the groups fed tucum-do-cerrado (Tuc; *p* = 0.001) and tucum-do-cerrado iron-enriched (TucFe) diets relative to the control group (*p* = 0.002), and the TuFe group also showed lower Ftl mRNA levels than the Fe group (*p* = 0.002; Figure 5).

## 4. Discussion

Our previous study performed *in vitro*, showed that tucum-do-cerrado, a fruit that is native to the Cerrado, has more phenolic compounds and higher antioxidant activity (AA) than the Red Delicious apple [18], which has a high known AA [26,27]. In the present study, we analyzed the effects of daily tucum-do-cerrado (pulp and peel) consumption on the oxidative stress in rats treated with an iron-enriched diet.

Despite the thin molecular regulation of intestinal iron absorption, the chronic consumption of an iron-enriched diet results in iron accumulation in tissues and, consequently, increases oxidative stress in tissues [16,28]. In the present study, Wistar rats were treated with an iron-enriched diet during 30 day, to induce oxidative stress. As expected, the iron-supplemented rats (Fe) showed an increase in liver, spleen and intestine iron concentration (Figure 1A,B) and a significantly increase of MDA level in liver (Figure 2A), besides an increase of serum iron levels and transferrin saturation (Table 2). Contrary to that obtained in the liver, the increase of iron concentration found in the spleen and intestine of iron-enriched rats, did not increase protein and lipid oxidative damage in these two tissues.

On the other hand, the addition of tucum-do-cerrado to the rodent’s diet (Tuc diet) significantly decreased oxidative stress in the spleen (Figure 2A,B) and increased the serum antioxidant capacity relative to the control group (Figure 3F). The decrease of biomolecules oxidative damage in the spleen of tucum-do-cerrado group suggests that this tissue may accumulate some of the antioxidant molecules of this fruit. Recently, Badria, *et al.* [29] demonstrated that supplementation of the curcumin polyphenol to iron-overloaded rats significantly decreased the levels of malondialdehyde and nitric oxide (NO) in spleen, suggesting an iron chelator property of curcumin. The improvement of spleen antioxidant capacity by tucum-do-cerrado is an important protection to this tissue since it is continuously exposed to high free iron content produced by the reticuloendothelial system during the recycling of the senescent red blood cells. The antioxidant effect of tucum-do-cerrado was also observed in the rats where the oxidative stress was induced by iron accumulation, once liver lipid peroxidation was lower and serum antioxidant capacity was higher in TucFe group relative to the Fe group. Furthermore, tucum-do-cerrado consumption abolished iron-induced changes in antioxidant enzymes activities in the kidney, as well as in the intestine, relative to the Fe group (Figure 3A,D). Pereira, *et al.* [30] verified that the consumption of tropical fruit juices reduced the activity of GPx, CAT and superoxide dismutase. The authors attributed this effect to the dietary antioxidants in the juice, which may have reduced the requirement for the antioxidant enzymatic function.

Considering the role of Nrf2 in the defenses of cells against oxidative stress and that consumption of fruits or fruit extracts attenuates oxidative stress by up-regulating Nrf2-dependent antioxidant enzymes [31,32,33], we investigated the effect of tucum-do-cerrado consumption on Nrf2 gene expression and antioxidant enzymes activity. In the present study, the increase of Nrf2 and its target genes, Hmox1 and Nqo1 transcripts levels in the liver of rats fed Tuc diet (Figure 4B,C), suggests that tucum-do-cerrado may induce liver Hmox1 and Nqo1 mRNA via Nrf2 up-regulation. A similar response was also observed in an early study performed in cells, where resveratrol up-regulates heme oxygenase-1 (HO-1) gene expression by activation of Nrf2 protein [31,32]. Peng, *et al.* [34] also showed that the polyphenol hydroxytyrosol from olive oil induces expression of several Nrf2 target antioxidant genes (HO-1, NQO1, Trx1, TrxR1, GCLC, and GCLM) and consequently protects neuronal cell against oxidative stress cell damage. The major polyphenol constituent of green tea, (−)-epigallocatechin-3-gallate (EGCG), increased HO1, NQO1, GSTm1, and GST hepatic mRNA levels in mice [35]. The up-regulation of hepatic Hmox1 and Nqo1 mRNA levels by tucum-do-cerrado (Tuc group) reinforces the hypothesis that tucum-do-cerrado augments the antioxidant capacity *in vivo*, once the enzyme HO-1 eliminates the potentially oxidant effect of free heme group, by degrading it [36,37] and the NQO1 reduces quinone compounds, preventing the formation of reactive oxygen species [38]. Corroborating with our results, Yang, *et al.* [39] showed that flavonoids, butein and phloretin, protect rat hepatocytes against tert-butylhydroperoxide-induced oxidative damage, through increasing the levels of HO-1 mRNA and protein. Contrary to that observed for Hmox1 and NQO1, no significant alteration was found in the activity of the four-antioxidant enzymes (catalase, GR, GPx and GST) activity in any studied tissues by tucum-do-cerrado consumption (Figure 3). However, tucum-do-cerrado consumption reduced the activity of the pro-oxidant enzyme, Nox, in the spleen (Figure 3).

Further, dietary iron overload also activates the Nrf2, and consequently genes involved in antioxidant defenses [13]. In the present study, the accumulation of the oxidant agent iron in the intestine of the rats treated with iron-enriched diet (Fe; Figure 1B) may have resulted in the up-regulation of Nrf2 gene expression in this tissue (Figure 4B), once Fe group showed higher Fe and Nrf2 mRNA levels compared to control group. Despite the high concentration of antioxidant compounds in tucum-do-cerrado fruit, as well as, the antioxidant effect of tucum-do-cerrado *in vivo* demonstrated by the reduced intestinal GPx activity and the high serum antioxidant capacity of TucFe rats relative to the Fe rats, the addition of tucum-do-cerrado to the iron supplemented diet (TucFe) failed to inhibit the increase of Nrf2 mRNA levels by iron (Figure 4). Unlike the present study, earlier studies have reported an increase in hepatic Nrf2 mRNA levels in iron-supplemented rats. These apparently controversial results rely upon some differences between treatments. Silva-Gomes, *et al.* [12] and Moon, *et al.* [13] treated mice with a diet supplemented with 2.0% carbonyl iron and 0.5% of 3,5,5-trimethyl-hexanoyl-ferrocene, respectively, in which iron content was substantially higher than that used in our study (350 mg of iron/kg of diet).

Due to the key role of hepcidin in the modulation of intestinal iron absorption and iron release from spleen [6], we investigated whether the antioxidant potential of tucum-do-cerrado is mediated by the modulation of Hamp expression, and consequently by tissues iron levels. According to the fine adjustment of iron homeostasis in the body, the accumulation of iron in the liver of iron-enriched rats (Figure 1), led to the strong up-regulation of liver hepcidin transcripts compared to control group (Figure 5). Unexpectedly, the consumption of tucum-do-cerrado diet (Tuc) significantly reduced the mRNA levels of Hamp in the liver (Figure 5), even though the total iron concentration in all studied tissues was not different compared to the control group. Further, tucum-do-cerrado inhibited the iron-induced hepatic hepcidin expression, despite the TucFe rats presented hepatic iron concentration similar to the Fe group. In addition, the spleen and intestine iron levels in the TucFe group were not different from the Fe group, and the levels of serum iron and transferrin saturation were significantly reduced in TucFe group (Table 2). We speculate that the down-regulation of Hamp expression by tucum-do-cerrado may be associated to a direct inhibitory effect of its phytochemicals compounds in the phosphorylation of transcription factors mothers against decapentaplegic homolog proteins 1/5/8 (SMAD 1/5/8) or signal transducer and activator of transcription 3 or 5 (STAT 3 or 5). Guan, *et al.* [40] observed in Huh7 cells treated with an extract of the Chinese medicinal plant *Caulis spatholobi* (rich in flavonoids) an inhibition of Hamp expression, even in the presence of increasing concentrations of BMP6. The authors demonstrated that the reduction in HAMP expression is mainly due to reducing the levels of phosphorylated SMAD 1/5/8 levels by CS extract, independently of BMP6 content. Similar results were also observed in rats treated with a polysaccharide from *Angelica sinensis* [41] or black soybean seed coat extract [42], the phytochemicals of these plants decreased hepcidin and phosphorylated SMAD 1/5/8 in a dose dependent manner. Therefore, it is possible that tucum-do-cerrado interferes in iron-dependent pathways at phosphorylation level of SMAD 1/5/8 or in iron-independent pathways triggered by IL-6 or erythropoietin.

Considering the high levels of polyphenols (flavanols: 717.6 mg catechin/100 g; yellow flavonoids: 42.3 mg/100 g and anthocyanins: 83.2 mg/100 g) identified in tucum-do-cerrado fruit [18], we speculate that one or more polyphenols of tucum-do-cerrado may exert an iron chelating or sequestering effect, and therefore, maintain iron unavailable in tissue. The maintenance of iron in a non-bioavailable form may have simulated iron depletion in hepatocytes, resulting in the inhibition of hepcidin expression. The decrease of hepatic Ftl mRNA levels in the rats treated with tucum-do-cerrado, independent of iron concentration in the liver (Tuc and TucFe groups), reinforces the suggestion that polyphenols of the tucum-do-cerrado may leave the iron unavailable for cell use. Usually, intracellular iron depletion leads to a decrease of the mRNA and protein ferritin levels [43]. Minear, *et al.* [44] demonstrated that curcumin (a polyphenol derived from a dietary spice used extensively in traditional Indian medicine) inhibits yeast growth by reducing the intracellular iron pool. The polyphenol curcumin penetrates in yeast cells, concentrates in the endoplasmic reticulum (ER) membranes and decrease intracellular iron availability. Together, these results strengthen the evidence that bioactive molecules of tucum-do-cerrado might have altered iron homeostasis, by reducing cellular iron availability and consequently ameliorates the antioxidant/oxidant status *in vivo.*

Considering that hepatic BMP6 modulates Hamp expression in response to liver iron concentration [45], we investigated if inhibition of Hamp by tucum-do-cerrado consumption involves BMP6 pathway. As expected, the accumulation of iron in the liver of iron-enriched group (Fe group), induced the Bmp6 gene expression in the liver, and consequently, may have up-regulated liver hepcidin transcripts compared to control group (Figure 5). A similar increase in hepatic Bmp6 gene expression was observed in the groups treated with tucum-do-cerrado (Tuc and TucFe). However, unlike observed in the Fe group, the iron concentration in the liver of Tuc group was similar to the control group, and the Hamp mRNA levels was reduced. In addition, the association of tucum-do-cerrado to the iron-enriched diet resulted in Bmp6 mRNA levels even higher than obtained for the Fe and Tuc groups; despite similar liver iron concentration and lower Hamp mRNA levels in relation to the Fe group. These results suggest that the induction of hepatic Bmp6 expression by tucum-do-cerrado is independent of tissue iron concentration, and can be directly modulated by polyphenols. Furthermore, these results also suggest that, the modulation of hepatic Hamp expression, by tucum-do-cerrado, is not mediated by the mechanism triggered by Bmp6.

## 5. Conclusions

In conclusion, the results evidence an *in vivo* antioxidant effect of tucum-do-cerrado, even in a condition of high concentration of iron, a potent oxidant agent. The antioxidant effect of tucum-do-cerrado may be associated to the chelate or sequester effect of one or more tucum-do-cerrado compounds that reduce iron availability in liver and down-regulate liver Hamp expression.

## Supplementary Materials

The following are available online at www.mdpi.com/link, Table S1: Dietary compositions, Table S2: Primer sequences used for Bmp6, Cat, Ftl, Hamp, Hmox1, Nqo1, Nrf2 and Actb real-time PCR assays.
