# Supplementary material for: Tucum-Do-Cerrado (Bactris setosa Mart.) Consumption Modulates Iron Homeostasis and Prevents Iron-Induced Oxidative Stress in the Rat Liver
Source: Nutrients. 2016 Feb 17;8(2):38. doi: 10.3390/nu8020038 (PMC4772024; doi:10.3390/nu8020038)
Supplement: Supplementary file 1 [file nutrients-08-00038-s001.docx]

Supplementary Material: Tucum-Do-Cerrado
[*Bactris setosa* Mart.] Consumption Modulates Iron Homeostasis and Prevents Iron-Induced Oxidative Stress in the Rat Liver

Adriana M. Fustinoni-Reis, Sandra F. Arruda, Lívia P. S. Dourado, Marcela S. B. da Cunha and Egle M. A. Siqueira

**Table S1.** Dietary compositions.

| **Components** | **Diets (g/kg)** | | | |
| --- | --- | --- | --- | --- |
|  | **CT** | **Fe** | **Tuc** | **TucFe** |
| Cornstarch | 397.5 | 397.5 | 369.6 | 369.6 |
| Starch (tucum-do-cerrado) | n.a. | n.a. | 27.8 | 27.8 |
| Protein (casein) | 200.0 | 200.0 | 198.3 | 198.3 |
| Protein (tucum-do-cerrado) | n.a. | n.a. | 1.7 | 1.7 |
| Dextrinized starch | 132.0 | 132.0 | 132.0 | 132.0 |
| Sucrose | 100.0 | 100.0 | 100.0 | 100.0 |
| Soybean oil | 70.0 | 70.0 | 69.7 | 69.7 |
| Fat (tucum-do-cerrado) | n.a. | n.a. | 0.3 | 0.3 |
| Fiber (celulose) | 50 | 50 | 47.5 | 47.5 |
| Fiber (tucum-do-cerrado) | n.a. | n.a. | 2.5 | 2.5 |
| Vitamin mix | 10.0 | 10.0 | 10.0 | 10.0 |
| Mineral mix without iron | 35.0 | 35.0 | 35.0 | 35.0 |
| L-cystine | 3.0 | 3.0 | 3.0 | 3.0 |
| Choline bitartrate | 2.5 | 2.5 | 2.5 | 2.5 |
| Iron | 0.035 | 0.350 | 0.035 | 0.350 |

Control diet (CT): AIN-93G rodent diet; iron-supplemented diet (Fe): AIN-93G rodent diet iron-enriched (350 mg of iron/kg diet, in the form of ferrous sulfate heptahydrate); tucum-do-cerrado diet (Tuc): AIN-93G diet containing 15% of tucum-do-cerrado (pulp and peel); iron-supplemented diet added of tucum-do-cerrado (TucFe): AIN-93G diet with iron supplementation (350 mg of iron/kg) and 15% of tucum-do-cerrado (pulp and peel). n.a.: not available.

**Table S2.** Primer sequences used for Bmp6, Cat, Ftl, Hamp, Hmox1, Nrf2, Nqo1 and Actb real-time PCR assays.

| **Gene** | **Primer Sequence (5′-3′)** | **Genbank Accession Number** | **Reference** |
| --- | --- | --- | --- |
| Bmp6 | GACAGCAGAGTCGCAATCG (forward) | NM_013107 | [1] |
|  | AGCTCACGTAAAGCTCATGC (reverse) |  |  |
| Cat | ACTCAGGTGCGGACATTC (forward) | NM_012520 | [2] |
|  | GGAGTTGTACTGGTCCAGAAGAGCC (reverse) |  |  |
| Ftl | CCTACCTCTCTCTGGGCTTCT (forward) | NM_022500 | [3] |
|  | CTTCTCCTCGGCCAATTC (reverse) |  |  |
| Hamp | TGATGCTGAAGCGAAGGA (forward) | NM_053469 | [4] |
|  | TGTGTTGAGAGGTCAGGAC (reverse) |  |  |
| Hmox1 | ATCGTGCTCGCATGAAC (forward) | NM_012580 | [5] |
|  | CAGCTCCTCAAACAGCTCAA (reverse) |  |  |
| Nrf2 | GAGACGGCCATGACTGAT (forward) | NM_031789 | [6] |
|  | GTGAGGGGATCGATGAGTAA (reverse) |  |  |
| Nqo1 | CAGCGGCTCCATGTACT (forward) | NM_017000 | [7] |
|  | GACCTGGAAGCCACAGAAG (reverse) |  |  |
| Actb | GTCGTACCACTGGCATTGTG (forward) | NM_031144 | [8] |
|  | CTCTCAGCTGTGGTGGTGAA (reverse) |  |  |

**References**

1. Ishizaki, N.; Kotani, M.; Funaba, M.; Matsui, T. Hepcidin expression in the liver of rats fed a
   magnesium-deficient diet. *Br. J. Nutr.* **2011**, *106*, 1169–1172.
2. Grigoryants, V.; Hannawa, K.K.; Pearce, C.G.; Sinha, I.; Roelofs, K.J.; Ailawadi, G.; Deatrick, K.B.; Woodrum, D.T.; Cho, B.S.; Henke, P.K.; *et al*. Tamoxifen up-regulates catalase production, inhibits vessel wall neutrophil infiltration, and attenuates development of experimental abdominal aortic aneurysms.
   *J. Vasc. Surg.* **2005**, *41*, 108–114.
3. Bulvik, B.; Grinberg, L.; Eliashar, R.; Berenshtein, E.; Chevion, M.M. Iron, ferritin and proteins of the methionine-centered redox cycle in young and old rat hearts. *Mech. Ageing Dev.* **2009**, *130*, 139–144.
4. Christiansen, H.; Sheikh, N.; Saile, B.; Reuter, F.; Rave-Frank, M.; Hermann, R.M.; Dudas, J.; Hille, A.; Hess, C.F.; Ramadori, G. X-irradiation in rat liver: Consequent upregulation of hepcidin and downregulation of hemojuvelin and ferroportin-1 gene expression. *Radiology* **2007**, *242*, 189–197.
5. Katavetin, P.; Inagi, R.; Miyata, T.; Shao, J.; Sassa, R.; Adler, S.; Eto, N.; Kato, H.; Fujita, T.; Nangaku, M. Erythropoietin induces heme oxygenase-1 expression and attenuates oxidative stress. *Biochem. Biophys.
   Res. Commun.* **2007**, *359*, 928–934.
6. Palsamy, P.; Subramanian, S. Resveratrol protects diabetic kidney by attenuating hyperglycemia-mediated oxidative stress and renal inflammatory cytokines via nrf2-keap1 signaling. *BBA Mol. Basis Dis.* **2011**, *1812*, 719–731.
7. Yamashita, Y.; Ueyama, T.; Nishi, T.; Yamamoto, Y.; Kawakoshi, A.; Sunami, S.; Iguchi, M.; Tamai, H.; Ueda, K.; Ito, T.; *et al*. Nrf2-inducing anti-oxidation stress response in the rat liver–New beneficial effect of lansoprazole. *PLoS ONE* **2014**, *9*, doi:10.1371/journal.pone.0097419.
8. Wang, Q.; Du, F.; Qian, Z.M.; Ge, X.H.; Zhu, L.; Yung, W.H.; Yang, L.; Ke, Y. Lipopolysaccharide induces a significant increase in expression of iron regulatory hormone hepcidin in the cortex and substantia nigra in rat brain. *Endocrinology* **2008**, *149*, 3920–3925.
